# HLA-DR monocyte antigen expression as predictors of outcome in patients with community-acquired infections presenting with fever

**DOI:** 10.1186/cc14126

**Published:** 2015-03-16

**Authors:** D Logothetis, E Giourou, I Starakis, A Lekkou, G Theodorou, M Leotsinidis, M Karakantza, C Gogos

**Affiliations:** 1 University of Patras, Patra, Greece

## Introduction

The aim of the present study was to evaluate the prognostic value of HLA-DR antigen expression in monocytes, in patients with community-acquired infections presenting with fever, as possible markers for the patients' final outcome.

## Methods

A total of 81 patients (males = 46; females = 35) presenting with fever >38°C to the emergency room (ER) of the Department of Internal Medicine of the Patras University Hospital were enrolled in the study during a period of 12 months. Sera for monocyte HLA-DR expression were obtained from the patients on admission (day 1) and on days 3, 7 or discharge/death. Results were expressed as percentages of HLA-DR-positive monocytes, calculated by the coexpression of CD14 and HLA-DR antigens in the total CD14^+ ^population. Additionally, the patients were evaluated using the Simplified Acute Physiology Score (SAPS-II), the Sequential Organ Failure Assessment (SOFA) and the Mortality in Emergency Department Sepsis (MEDS) score on the same days while all the indicated clinical, laboratory and imaging procedures as required for fever's differential diagnosis were followed. A questionnaire regarding demographic characteristics, comorbidities, medications used and patients' survival was also completed. All statistical analyses were performed using SPSS v.21.

## Results

Lower mean HLA-DR monocyte antigen expression percentages were significantly correlated to lower Glasgow Scale scores on all days of measurement. HLA-DR expression was significantly negatively correlated to MEDS, SOFA and SAPS-II scores whereas patients who developed sepsis, severe sepsis, septic shock and MODS had significantly lower HLA-DR values compared with the ones who did not. HLA-DR expression on day 1 was lower in patients who would develop SIRS and/or sepsis on days 3 and 7 (*P *< 0.01). Additionally, HLADR expression was significantly decreased in nonsurvivors (*n *= 33) compared with survivors (*n *= 48), whereas lower HLA_DR expression was correlated to longest duration of hospital stay at all time points (*P *< 0.01).

## Conclusion

Monocyte HLA-DR appears to be an early indicator for survival and infection progression and therefore it can be used as a predictive marker for the final outcome of patients presenting in ER departments with fever.

